# Weighted averaging in spectroscopic studies improves statistical power

**DOI:** 10.1002/mrm.26615

**Published:** 2017-01-26

**Authors:** Jack J. Miller, Lowri Cochlin, Kieran Clarke, Damian J. Tyler

**Affiliations:** ^1^ Department of Physiology, Anatomy & Genetics University of Oxford Oxford UK; ^2^ Department of Physics, Clarendon Laboratory University of Oxford Oxford UK; ^3^ Present address: PulseTeq UK Ltd, 64‐66 High Street Chobham, Surrey GU24 8AA

**Keywords:** 13C spectroscopy, 31P spectroscopy, biostatistics, hyperpolarized 13C, magnetic resonance spectroscopy, phosphorus MRS, uncertainty analysis

## Abstract

**Purpose:**

In vivo MRS is often characterized by a spectral signal‐to‐noise ratio (SNR) that varies highly between experiments. A common design for spectroscopic studies is to compare the ratio of two spectral peak amplitudes between groups, e.g. individual PCr/*γ*‐ATP ratios in ^31^P‐MRS. The uncertainty on this ratio is often neglected. We wished to explore this assumption.

**Theory:**

The canonical theory for the propagation of uncertainty on the ratio of two spectral peaks and its incorporation in the Frequentist hypothesis testing framework by weighted averaging is presented.

**Methods:**

Two retrospective re‐analyses of studies comparing spectral peak ratios and one prospective simulation were performed using both the weighted and unweighted methods.

**Results:**

It was found that propagating uncertainty correctly improved statistical power in all cases considered, which could be used to reduce the number of subjects required to perform an MR study.

**Conclusion:**

The variability of in vivo spectroscopy data is often accounted for by requiring it to meet an SNR threshold. A theoretically sound propagation of the variable uncertainty caused by quantifying spectra of differing SNR is therefore likely to improve the power of in vivo spectroscopy studies. Magn Reson Med 78:2082–2094, 2017. © 2017 The Authors Magnetic Resonance in Medicine published by Wiley Periodicals, Inc. on behalf of International Society for Magnetic Resonance in Medicine. This is an open access article under the terms of the Creative Commons Attribution License, which permits use, distribution and reproduction in any medium, provided the original work is properly cited.

## INTRODUCTION

Typically, in vivo magnetic resonance spectroscopy (MRS) studies aim to determine an underlying biological difference between groups of subjects by comparing quantities of interest that are computed from spectra acquired from multiple individuals. For example, the ratio of the amplitude of a small molecule spectral peak to a concentration standard is commonly computed per individual, such as the phosphocreatine to ATP (PCr/ATP) ratio in cardiac ^31^P spectroscopy 1, or the ^13^C‐bicarbonate to [1‐^13^C]pyruvate ratio in hyperpolarized ^13^C MRS 2. Additionally, MRS studies are typically characterized by a highly variable signal‐to‐noise ratio between individuals and over time, resulting in acquired data that is of variable quality. Most spectroscopic quantification algorithms such as AMARES 3, VARPRO 4, or Bayesian methods 5, 6 return an estimate of the measure of uncertainty in the resultant fitting of spectral peaks: AMARES returns the Cramér‐Rao Lower Bound (CRLB) of amplitude uncertainty directly 7, 8; for Bayesian methods such a measure of uncertainty would be the width of the posterior probability distribution function at the end of the algorithm 9. It is often the case that this intrinsic variability of spectral data is taken into account by imposing a quality minimum on the acquired spectra; for example, by requiring the signal‐to‐noise ratio of an acquired spectrum to be above an arbitrary threshold, and then subject only the population of data that are ‘good enough’ to further analysis 10.

We hypothesized that the correct propagation of uncertainty throughout the analysis of spectroscopy data would improve accuracy, precision, and statistical power. Briefly, a simple, straightforward and canonical method to propagate uncertainty from spectroscopic quantification is presented, and illustrated with numerical simulations. The statistical framework we propose is broadly similar to that used in other physical sciences 11. A retrospective analysis of existing data was then performed based on published work in two distinct biological settings that have been extensively validated by other techniques. In all cases, the analysis proposed allowed the same conclusion to be drawn with greater certainty, or, alternatively, would necessitate fewer subjects in each group be scanned in order to perform a study of identical statistical power. The method presented is additionally broadly applicable.

## THEORY

Fitting algorithms, such as AMARES, typically return an estimated parameter of interest for an individual spectral line in an individual's MRS spectrum (such as its frequency location, or amplitude), together with a measure of the uncertainty on this measurement, usually the Cramér‐Rao Lower Bound on its variance. As spectroscopic estimates are obtained by measurements based on a large number of discrete points, and the noise in MR experiments is approximately white, it is appropriate to consider each one of these estimates as random variables, drawn from a normal distribution with mean defined by the value of the estimator and variance defined by its estimate of uncertainty. Strictly, noise in MR is white in both the real and imaginary parts; therefore, magnitude data is Rician distributed, rather than normally distributed, and therefore one has to consider whether the quantity returned by a spectral peak fitting algorithm is constrained to be greater than zero, e.g. by returning magnitude‐only information. The two distributions are the same in the high SNR limit, practically where SNR > 2 12. The approximation of normality (taken subsequently here) is therefore valid in either this SNR limit, if the fitting algorithm used returns quantities that can be negative, or if it considers only real or imaginary data.

This work details the propagation of the CRLB uncertainty throughout typical analyses performed in MR studies through weighted averaging. Note that the Cramér‐Rao Lower Bound on the variance of an estimated parameter is exactly that: a *lower* bound on the uncertainty on a spectral peak estimator. Whilst the ‘true’ uncertainty on the quantity of interest may be higher, the use of the CRLB as a weight is attractive, as (a) it is readily obtainable directly from commonly used spectral quantification algorithms; and (b) it represents the minimum uncertainty in spectral estimates of the data considered; no unbiased estimator can outperform it. Owing to the reciprocal relationship between this uncertainty and its subsequent weighted expression, it is therefore appropriate to consider the CRLB as a ‘worst‐case’ scenario for the impact that a particular data point would have on the estimates of the population as a whole. As the estimation of the CRLB is computationally straightforward and routine in the context of spectral fitting, and well understood by the community to be increased if spectral quality is poor, we propose that its use as a weight is therefore wholly appropriate.

As the overall absolute detected magnitude of spectral peaks is highly dependent on experimental conditions such as the coil‐subject distance, it is often desirable to calculate functions of the measured data, such as the ratio of the amplitude of two different spectral peaks, or the sum of several uncertain peaks. In general, such peak amplitudes within a spectrum may be correlated with coefficient ρ∈[0, 1], the determination of which is ultimately an experimental question: *ρ* can be obtained from the Fisher information matrix for the fitting procedure in question $F_{ij}$ (from which the CRLB itself is derived as $\sqrt{F_{ii}^{-1}}$), as $\rho_{ij}=F_{ij}^{-1}/\sqrt{F_{ii}^{-1}F_{jj}^{-1}}$ between peak amplitudes *i* and *j* 13.

In general, there is no exact expression for functions of normally distributed random variables, and numerical (Monte Carlo) simulations have to be performed to determine the shape of the resulting distribution.

### Ratios

In the special, but common, case of two random variables, it is often under‐appreciated that the resulting distribution is in general not normal, but rather described by a ratio distribution, with a width that is reduced if the variables are correlated 14, 15. Analytic forms for the width of this distribution can be computed either exactly or under the approximation that uncertainty is small.

### Small Errors

Consider two peaks from an individual spectrum, estimated to be of magnitude *x* and *y* with associated quantification uncertainties *σ_x_* and *σ_y_*. If $\sigma_{x}/x\ll 1$ and $\sigma_{y}/y\ll 1$, and neither *x* nor *y* are close to zero, then the distribution of their ratio is very well approximated by the normal distribution with mean μ=x/y 16. In this case, the width of the distribution (and hence the uncertainty on *x*/*y* for subject *i*) is approximately

(1) $$\sigma_{i}\approx \frac{x}{y}\sqrt{{\left(\frac{\sigma_{x}}{x}\right)}^{2}+{\left(\frac{\sigma_{y}}{y}\right)}^{2}-2\rho \frac{\sigma_{x}}{x}\frac{\sigma_{y}}{y}}.$$

Equation (1) can be derived from the multivariate Taylor series expansion for the function $f(x,y)=\frac{x}{y}$, and reduces to the common ‘errors in quadrature’ form commonly encountered if *ρ* = 0. The case where the error on measurement is large compared to the measurement made (i.e. where $\sigma_{x}/x$ or $\sigma_{y}/y$ are not much less than one) is more complex, and discussed subsequently.

### Large Errors

Alternatively, consider the case where the estimated error on a peak amplitude in an NMR spectrum is large, and where the estimated $\sigma_{x}/x$ is not ≪1 (i.e. approximately 0.1 or greater). If we consider the ratio of two peaks where the error is large, the resulting quantity has a Gaussian ratio distribution, described by Fieller, Hinkley and others 15, 17. We propose that the returned CRLB from the quantification of each peak can be treated as exactly defining a normal probability density function (PDF) for the quantity of interest for that peak. The resulting distribution for the ratio $w=(x∼N(\mu_{x},\;\sigma_{x}))/(y∼N(\mu_{y},\;\sigma_{y}))$ where *x* and *y* have a correlation coefficient *ρ*, has 15 an analytic PDF *f*(*w*) defined in the appendix.

**Figure 1:**
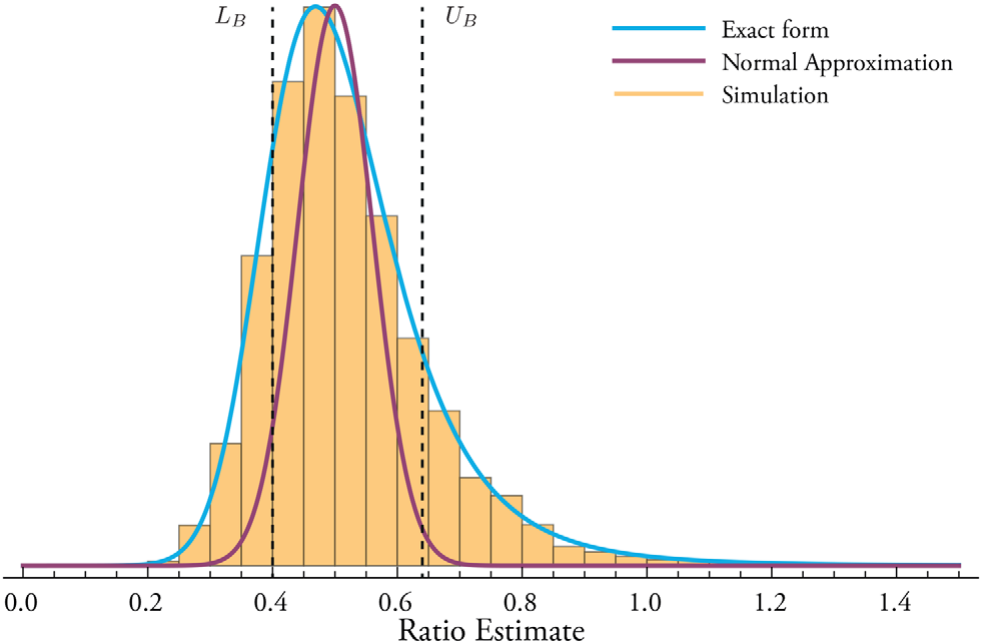
The PDF for the ratio of two normally distributed random variables, with $\mu_{x}=1,\;\sigma_{x}=0.2,\;\mu_{y}=2,\;\sigma_{y}=0.5$ and correlated with ρ=0.5. Plotted here on common axes are the PDFs from the normal approximation (purple) given in Eq. [1], and the true analytic form stated in Eq. [A1] *et passim* (blue). The median for both distributions is the ratio of means (i.e. $\frac{1}{2}$), but the means are distinct at 0.5 and 0.525. The orange boxes show a histogram of counts ( $n=10^{4}$) from the division of two normally distributed random variables generated with the parameters as given. The choice of values here is illustrative of a ‘moderately pathological’ case, where the normal approximation is clearly not appropriate, and should be contrasted to the ‘highly pathological’ case where $\mu_{x}=\mu_{y}=0$ and $\sigma_{x}=\sigma_{y}=1$ (i.e. a Cauchy distribution where no mean and variance exist) and the ‘physiological’ case where both means are far from zero compared to *σ*. Dotted black lines denote the lower and upper bounds $\left(L_{B},\;U_{B}\right)$ at the “ 1σ” level, derived through the use of Fieller's theorem as stated in Eq. [2].

In general, this distribution is unimodal, not symmetric, and approaches the Cauchy distribution (also known as the Briet–Wigner distribution) as *μ_x_* and *μ_y_* tend to zero, and *σ_x_*, *σ_y_* tend to one. The Cauchy distribution does not have a defined mean or variance. An illustrative example of the analytic form of the Ratio PDF is provided in Figure 1, which shows both *f*(*w*) (as given by Eqs. [A1] to (A5)), and the approximate normal quantity as given by Eq. (1). If both means are far from zero, and the uncertainty in measurements is small, the quantity described in Eq. (1) asymptotically tends to Eq. (A1). Note that it is far easier to numerically evaluate the approximate form Eq. (1) as opposed to Eqs. [A1] to (A5), which frequently equate to the product of exponentially large and small quantities and hence issues arising from finite numerical precision cannot be neglected.

Note additionally that the ratio distribution *f*(*w*) defined above may be decidedly non‐normal, but that the central limit theorem ensures that the arithmetic mean of such ratio samples will be normally distributed in the limit that the number of samples becomes large, independent of this skew.

Several approaches to computing confidence limits on the ratio distribution have been historically proposed. Such confidence intervals are not guaranteed to exist as *μ_x_* and *μ_y_* tend to zero, reflecting the arbitrarily large increase in uncertainty arising from division by a number arbitrarily close to zero. Fieller's theorem allows for the computation of confidence limits on the ratio distribution, and can be derived from geometric arguments about the density of probability, and states that the lower and upper confidence limits (*L_B_*, *U_B_*) on the ratio w=x/y are given by 16, 17

(2) $$(L_{B},U_{B})=\frac{1}{\mu_{y}^{2}-t_{q}^{2}\sigma_{y}^{2}}((\mu_{y}\mu_{x}-t_{q}^{2}\sigma_{xy})\pm \sqrt{{\left(\mu_{y}\mu_{x}-t_{q}^{2}\sigma_{xy}\right)}^{2}-\left(\mu_{y}^{2}-t_{q}^{2}\sigma_{y}^{2}\right)\left(\mu_{x}^{2}-t_{q}^{2}\sigma_{x}^{2}\right)}),$$

provided that *μ_x_* is not expected to be close to zero, and where $\sigma_{xy}=\rho \;\sigma_{x}\sigma_{y}$. Formally, $t_{q}^{2}$ is the *t*‐statistic at the chosen significance level *α*, i.e. the value of the inverse cumulative *t* probability distribution function with *n* – 1 degrees of freedom at $q=1-\frac{\alpha }{2}$. In the context of spectroscopy performed on a large number of points, the estimation of uncertainty is free from sampling effects and it is appropriate to take n→∞, such that the *t*‐distribution tends to the normal one. If the variances are small and the denominator is “far” from zero, this expression is quantitatively similar to the familiar expression for the uncertainty on a ratio, Eq. (6) 17.

It is proposed that the width of the confidence interval defined above, $U_{B}-L_{B}$, at “ 1σ”, i.e. with $q=1-\text{Erf}\left(\frac{1}{\sqrt{2}}\right)$ is an appropriate weight to use for subsequent calculations provided that *μ_x_* is not expected to be close to zero. This choice of *q* provides, *t_q_* = 1 as n→∞ by definition, and both upper and lower bounds derived are illustrated in Figure 1. As a consequence, an appropriate weight $``\sigma_{i}^{\prime }\prime$ to consider for subsequent estimation of population properties of the mean can be constructed and simplified at “ 1σ” considerably, to form

(3) $$\sigma_{i}=U_{B}-L_{B}=\frac{2}{\mu_{y}^{2}-\sigma_{y}^{2}}\sqrt{\mu_{x}^{2}\sigma_{y}^{2}+\mu_{y}^{2}\sigma_{x}^{2}-2\mu_{x}\sigma_{xy}\mu_{y}-\sigma_{x}^{2}\sigma_{y}^{2}+\sigma_{xy}^{2}}.$$

Given the weight specified above, the rest of the analysis of such data would then proceed from Eq. (5) onwards.

### Sums

In contrast to the ratio of peaks as discussed above, the uncertainty on a sum or difference of a set of uncertain spectral peaks is analytically straightforward, as the sum of normally distributed variates is itself normal. Therefore, given a set $X_{1},\;X_{2},\;X_{3},\ldots =X_{i}$ of spectral peaks with means $\mu_{x},\;\mu_{y},\;\mu_{z},\ldots =\mu_{i}$ and CRLBs $\sigma_{x}^{2},\;\sigma_{y}^{2},\;\sigma_{z}^{2},\ldots =\sigma_{i}^{2}$, it can be shown that the sum $a_{1}X_{1}+a_{2}X_{2}+a_{3}X_{3}+\ldots =\sum_{i=1}^{n}a_{i}X_{i}$ (for some real numbers *a_i_*) is normally distributed with mean $\sum_{i=1}^{n}a_{i}\mu_{i}$ and variance

(4) $$\sigma_{X_{1}+X_{2}+X_{3}+\ldots }^{2}=\sum_{i=1}^{n}a_{i}^{2}\sigma_{i}^{2}+2\sum_{i<j1\leq j\leq n}^{n}\left(a_{i}a_{j}\text{Cov}(X_{i},X_{j})\right).$$

In the case where *n* = 2, this variance simplifies to the form $\sigma_{x+y}^{2}=a_{1}^{2}\sigma_{x}^{2}+a_{2}^{2}\sigma_{y}^{2}+2a_{1}a_{2}\rho \sigma_{x}\sigma_{y}$. Such sums are commonly encountered in MRS where the *a_i_* above are often equal to unity, e.g. when summing choline and creatine from a set of several peaks in proton spectroscopy, or providing a total ATP measure from the *α*‐, *β*‐ and *γ*‐ATP peaks in phosphorus spectroscopy. Should it then be required to take ratios to this uncertain sum, the derived uncertainty and mean should then themselves be treated as returned estimates for an individual peak, and the analysis proceed as for the ratio sections above.

### Population Comparisons

An elementary statistical result (derived in the Online Supporting Information) is that an unbiased estimator for the mean ( $\bar{r}$) *of the population* of these uncertain functions of measurements are given by the weighted sum

(5) $$\bar{r}=\frac{\sum_{i}^{n}\frac{\mu_{i}}{\sigma_{i}^{2}}}{\sum_{i}^{n}\frac{1}{\sigma_{i}^{2}}},$$

and the variance of the population, given the uncertainty on each measurement, is best estimated by

(6) $${\bar{\sigma }}_{r}^{2}=\left(\sum_{i}^{n}\frac{1}{\sigma_{i}^{2}}\right){\left({\left(\sum_{i}^{n}\frac{1}{\sigma_{i}^{2}}\right)}^{2}-\sum_{i}^{n}{\left(\frac{1}{\sigma_{i}^{2}}\right)}^{2}\right)}^{-1}\sum_{i=1}^{n}\frac{{\left(\mu_{i}-\bar{r}\right)}^{2}}{\sigma_{i}^{2}},$$

where *n* is the number of measurements made. Hence, given two populations of such ratios of measurements, *r*_1_ and *r*_2_ with *n*_1_ and *n*_2_ elements in each, it is often desirable to perform statistical tests between the groups. The weighted expressions Eqs. (5) and (6) are hence the appropriate quantities to use in such comparisons. For example, in order to perform the standard unequal variance *t*‐test (Welch's *t*‐test) one can compute the quantity

(7) $$t=\frac{{\bar{r}}_{1}-{\bar{r}}_{2}}{\sqrt{\frac{{\bar{\sigma }}_{r1}^{2}}{n_{1}}+\frac{{\bar{\sigma }}_{r2}^{2}}{n_{2}}}},$$

which approximately follows a *t*‐distribution with

(8) $$d=\frac{{\left(\frac{{\bar{\sigma }}_{r1}^{2}}{n_{1}}+\frac{{\bar{\sigma }}_{r2}^{2}}{n_{2}}\right)}^{2}}{{\left({\bar{\sigma }}_{r1}^{2}/n_{1}\right)}^{2}/(n_{1}-1)+{\left({\bar{\sigma }}_{r2}^{2}/n_{2}\right)}^{2}/(n_{2}-1)}$$

degrees of freedom. Therefore, in the Frequentist hypothesis testing framework, we can reject the null hypothesis that the two populations considered have the same mean if

(9) p=2×(1−pt(|t|, d))

is less than some chosen significance level (e.g., 0.05), where pt(|t|, d) is the cumulative *t*‐distribution function.

### Summary

**Figure 2:**
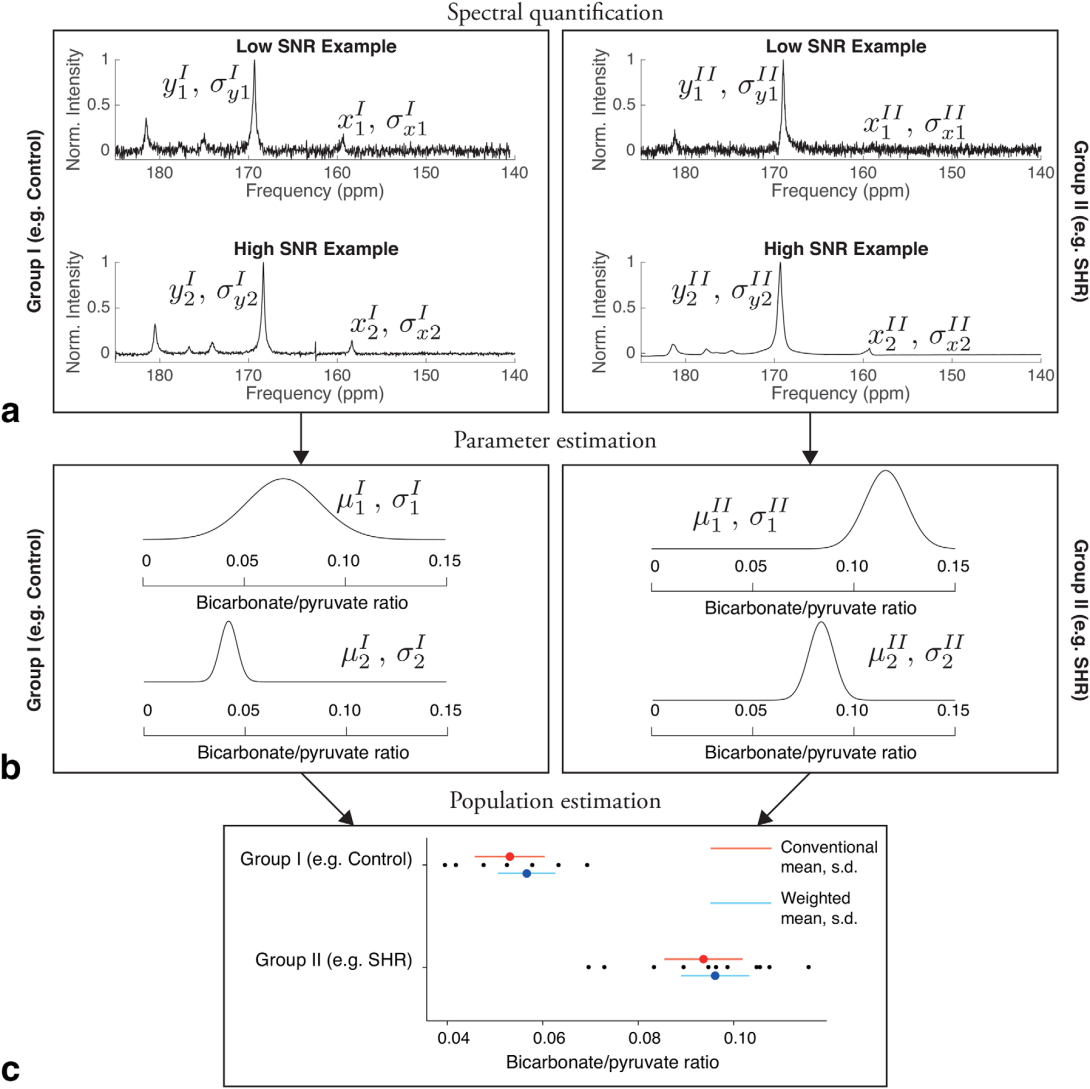
An overview of the proposed analysis method. **a**: Not all NMR spectra are acquired equal. For an individual NMR spectrum, quantification algorithms, such as AMARES, therefore return both an estimated parameter for a peak of interest, such as its amplitude *x_i_* or *y_i_*, together with an estimate of that uncertainty *σ_x_* or *σ_y_*. It is often the case that experiments are planned in which differences in f(x, y) are investigated between two groups of subjects, such as the ratio of peak amplitudes *x*/*y* (which here is visible hyperpolarized bicarbonate/pyruvate). **b**: Therefore, it is necessary to consider the uncertainty *σ* in the resulting quantity, which has an expected value *μ*. One therefore obtains two biologically separate populations of both measurements and uncertainties. Therefore, one can compute estimates for the mean and standard deviation of these measurements, as illustrated in **c** for the example case of the SHR study data. Here, the location of both the mean and standard deviation are shown together with the data for both the conventional analysis method (red) that ignores uncertainty, and the proposed technique (blue). The length of the horizontal red or blue bar on the population denotes its estimated standard deviation (s.d.), with the centre dot denoting the (weighted) mean. Note the apparent reduction in population variability obtained due to the propagation of measurement uncertainty, which would in turn improve statistical power.

This process assumes that different groups of multiple individuals undergo MRS in order to obtain spectra, of varying quality between experiments, that have more than one peak. Ultimately, the biological quantity of interest is often approximated by the ratio of two peak amplitudes, or ratios to their sum. As graphically summarized in Figure 2, the proposed process is as follows:

- For each individual *i* in each group, quantify the measured spectrum and obtain estimates of peak amplitudes *x_i_* and *y_i_* together with associated uncertainties *σ_x_* and *σ_y_* (Fig. 2a).
- Compute the corresponding individual quantity of interest, $f_{i}(x_{i},\;y_{i})$ which for the sake of illustration here is $\mu_{i}\overset{\text{def}}{=}x_{i}/y_{i}$ (Fig. 2b).
- Compute also the corresponding uncertainty on *μ_i_*, denoted *σ_i_* from either Eq. (1) or 3 depending on the magnitude of *x*, *y*, *σ_x_* and *σ_y_*. (Fig. 2b) Note that corresponding expressions for functions of peaks other than *x*/*y* can be readily obtained. For example, the distribution calculator Caladis (http://www.caladis.org) has been developed with the explicit aim of graphically illustrating these phenomena in a biological setting 18, 19.
- Hence, take the obtained two sets of measurements of quantities of interest in either group, e.g. arbitrarily called $\left\{\mu_{1}^{A},\;\sigma_{1}^{A},\ldots ,\mu_{n_{A}}^{A},\;\sigma_{n_{A}}^{A}\right\}$ and $\left\{\mu_{1}^{B},\;\sigma_{1}^{B},\ldots \mu_{n_{B}}^{B},\;\sigma_{n_{B}}^{B}\right\}$ for two groups *A* and *B* (with *n_A_* and *n_B_* members in each), and compute summary statistics for each group: the means of the groups ${\bar{r}}_{A}$ and ${\bar{r}}_{B}$ as per Eq. (5), and their variance, ${\bar{\sigma }}_{A}^{2}$ and ${\bar{\sigma }}_{B}^{2}$, as per Eq. (6) (Fig. 2c).
- Finally, perform appropriate statistical tests between the groups as required. As an example, an unequal variance *t*‐test (also known as Welch's *t*‐test) is described in Eqs. [7] to (9).

## METHODS

### Simulations

Two populations each containing *n* = 300 spectra were simulated consisting of two peaks, nominally called “PCr” and “ATP”. One population was nominally “healthy” in which the population PCr/*γ*‐ATP peak ratio was taken to be 2/1.025≈1.95, and one nominally “diseased', where PCr/*γ*‐ATP was 2/1.25=1.6. These values correspond approximately to reported values of the human PCr/*γ*‐ATP in healthy individuals and those with dilated cardiomyopathy 20. Additionally, two simulated sets of six of ^31^P cardiac MRS spectra were generated with chemical shift separations corresponding to 11.7 T, as an illustrative example of the effect of weighted averaging on a typical study by recapitulating a normal experimental situation. The linewidth of resonances were constant, and set appropriately based on measured values from previous experiments at 11.7 T (where *γ*‐ATP linewidth 67 ± 15 Hz, PCr linewidth 48 ± 16 Hz; values mean ± standard deviation, *n* = 6).

For each spectrum, a varying amount of white noise was added to the time domain real and imaginary FID. The amplitude of noise added was itself normally distributed, and was intended to reflect experiment‐to‐experiment variability in SNR. Expressed as a percentage of the maximum time domain signal intensity for each individual spectrum, the noise level *n* was distributed as either n∼N(μ=15 %,  σ=5%), to provide an example of highly uncertain data, or as n∼N(μ=1%,  σ=0.25%) to provide an example of higher quality spectra. As a result, 1206 spectra were generated in total: “healthy” and “diseased” populations with broadly good or bad SNR.

After generation, spectra were individually quantified using a custom MATLAB implementation of the AMARES algorithm 3, 21. Estimates for both peak amplitudes and measures of uncertainty on those estimates were stored, and were analyzed as outlined in the Theory section above, under the either the “small errors” or “large errors” regime as appropriate. Spectra were simulated and quantified in MATLAB. All statistical analysis was carried out in R 22.

Summary statistics and histograms were generated from the two populations of spectra obtained. A “bootstrapping” analysis was performed on the simulated populations, in which samples were drawn uniformly and the mean, median and weighted mean estimated. This process allows for the estimation of the sampling distribution of these estimators, and illustrates the effect of weighted averaging.

Additionally, for the illustrative example and subsequent re‐analyses, the Hedges' $g^{*}$ effect size for the difference between these two groups was computed as outlined previously 23, i.e. as

(10) $$g^{*}=\frac{\Gamma \left(\frac{n_{1}+n_{2}-2}{2}\right)}{\Gamma \left(\frac{n_{A}+n_{B}-3}{2}\right)\;\sqrt{\frac{n_{A}+n_{B}-2}{2}}}\frac{{\bar{r}}_{A}-{\bar{r}}_{B}}{s^{*}},$$

where Γ(x) is the gamma function, $\bar{r}$ denotes the population mean of the ratio quantity of interest, and $s^{*}$ is the estimated pooled standard deviation of the two groups with *n_A_* and *n_B_* members in each, defined as

(11) $$s^{*}=\sqrt{\frac{(n_{A}-1){\bar{\sigma }}_{A}^{2}+(n_{B}-1){\bar{\sigma }}_{B}^{2}}{n_{A}+n_{B}-2}},$$

with $\bar{\sigma }$ the estimated population variance. Power was computed by the ‘pwr’ package in R. 24 for both the conventional unweighted and the weighted population estimates of $\bar{r}$ and $\bar{\sigma }$.

### Retrospective Analysis

#### Hyperpolarized Spectra

The authors of a previously published study using hyperpolarized [1‐^13^C]pyruvate MRS to investigate myocardial metabolism in the spontaneously hypertensive rat (SHR) were contacted, and provided original data for re‐analysis. As described in Dodd et al. 25, ^13^C spectra were obtained every second following the injection of hyperpolarized [1‐^13^C]pyruvate into SHR rats who possess a series of genetic mutations that result in a spontaneously hypertensive phenotype, and from a set of age and sex matched wild‐type controls. For each rat, the ratio of the ^13^C‐bicarbonate to [1‐^13^C]pyruvate amplitudes of the summed hyperpolarized spectra was considered to be an appropriate normalized measure of pyruvate dehydrogenase (PDH) flux, and was computed by the simple ratio of amplitudes as quantified by AMARES after summation of the spectral data over time. Such analyses are appropriate, as the metabolite (here ^13^C‐bicarbonate) to [1‐^13^C]pyruvate ratio computed in this fashion has been shown to be linearly proportional to the biological rate constant of interest ( $k_{\text{Pyr}\to \text{Bic}}$) 26. In the study of Dodd et al., the apparent rate of ^13^C‐bicarbonate production was increased by 85% in the hypertensive animals compared to controls, corresponding to an inborn genetic up‐regulation of PDH activity. This increase was subsequently verified through extensive ex vivo biochemical techniques to determine PDH activity and protein expression of PDH regulatory enzymes that occurred with the development of cardiac hypertrophy.

For the reanalysis, temporally summed spectra were quantified by AMARES and were analyzed as outlined in the Theory section, under the “small errors” regime. The validity of the assumptions detailed were tested; despite the variability in SNR inherent in hyperpolarized MRS, the ratio of peak uncertainties to amplitudes was typically less than $10^{-2}$. Statistical power was additionally computed as specified previously in Eqs. [10] and (11), with the effect size inferred for both analysis techniques from all available spectra.

#### Human ^31^P Cardiac MR Spectra

Similarly, in order to provide an appropriate example of phosphorus spectra of varying SNR, the authors of a previously published study investigating the cardiac PCr/ATP ratio of diabetic patients were contacted, and provided data for re‐analysis. As described in Levelt et al. 27, ^31^P spectra were obtained from the mid‐ventricular septum of 31 patients with type two diabetes mellitus (T2DM, as diagnosed according to World Health Organisation guidelines) and matched controls through a Chemical Shift Imaging spectroscopic sequence with outer volume suppression to prevent spectral contamination from skeletal muscle, blood and hence reduce inter‐subject variation 28. Subjects were scanned under two conditions: at rest, and during exercise in the scanner bore provided by leg motion and knee flexion resisted by sets of 2.5 kg weights. Spectra were acquired on a 3 T Siemens Tim Trio according to the principles of the Declaration of Helsinki, with approval from the UK NHS Health Research Authority Research Ethics Committee (REC Ref. 13/SW/0257). Levelt et al. additionally further characterized the hearts by quantifying oxygen availability (through BOLD) and myocardial perfusion at rest and during adenosine stress, the presence or absence of coronary arterial disease through contrast enhanced CT, and cardiac function through an extensive number of CMR studies. It was found that the diabetic heart was energetically impaired, with a poor myocardial perfusion reserve, a decrease in oxygen consumption, and a decrease in myocardial PCr/ATP ratio, with a reported mean PCr/ATP ratio of 1.74±0.26 at rest compared to 1.54 ± 0.26 during exercise (mean ± SEM).

For the subsequent reanalysis, it was only possible to obtain data with permission for reuse from 22 of the 31 T2DM patients considered in the initial study, and from 28 matched controls corresponding to the initial rest arm of the study. Spectra were quantified with AMARES, with the estimated returned CRLB on peak amplitudes used as outlined in the Theory section, under the “small errors” regime. As the ratio of PCr/total ATP was considered by Levelt et al., the uncertainty in quantification on each of the three ATP peaks was propagated appropriately, by canonical expressions obtained via multivariate Taylor series expansion similar to Eq. (1) (i.e. “in quadrature”). Statistical power was additionally computed as specified previously in Eqs. [10] and (11), with the effect size inferred for both conventional, unweighted and weighted techniques from all available spectra.

## RESULTS

### Simulation

**Figure 3:**
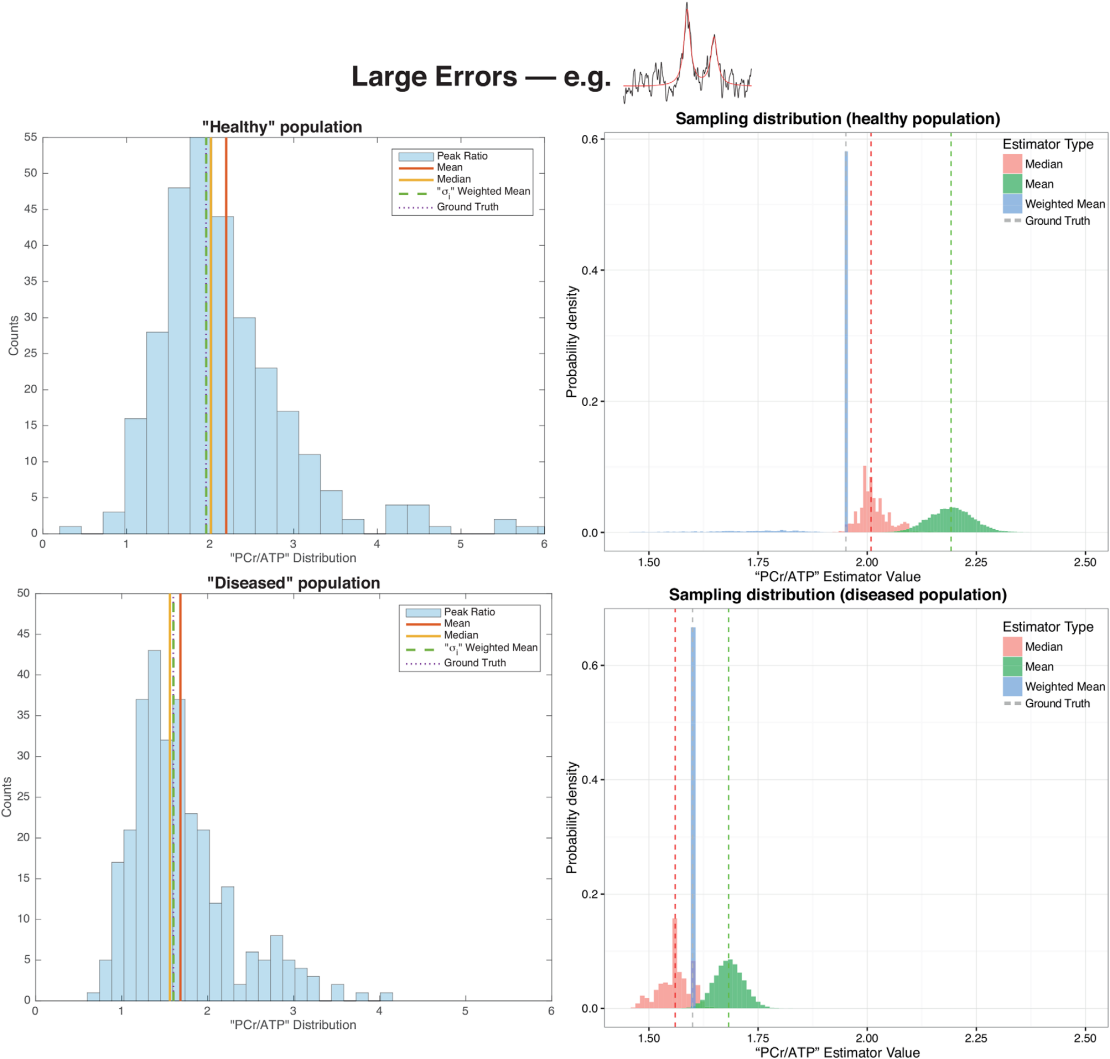
The distribution of apparent peak ratios for the two populations of uncertain spectra simulated under the “large errors' regime. Note that the distribution is not symmetric, and accordingly the mean, median and weighted mean differ from the ‘ground truth’ values used to generate the spectra. The sampling distribution of each estimator is also shown, as obtained through a bootstrap analysis of the simulated data.

**Figure 4:**
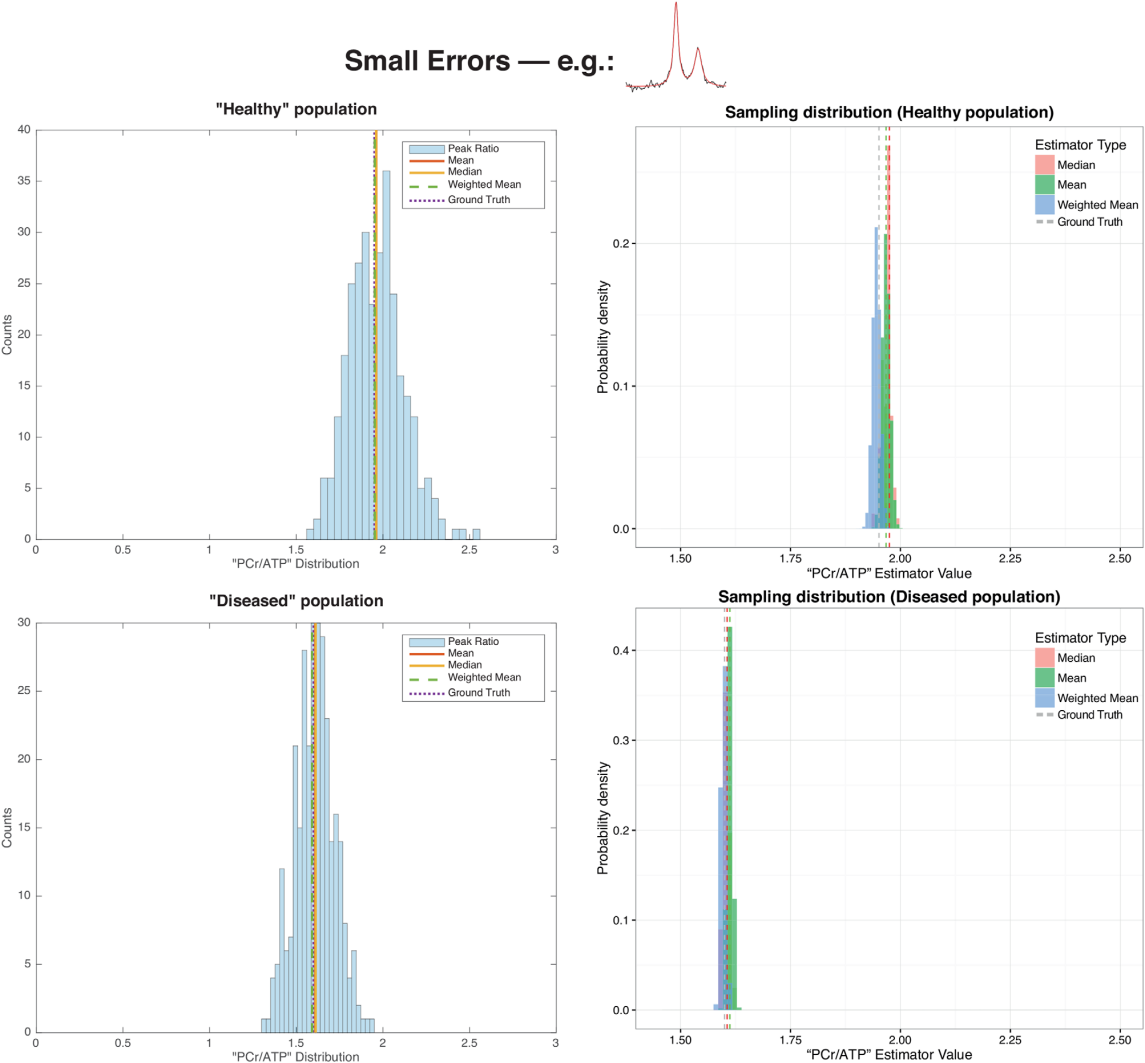
The distribution of apparent peak ratios for the two populations of spectra simulated under the “small errors” regime, with high SNR. In contrast to the large errors case, the distribution is more symmetric, and accordingly the sampling distribution of all estimators is less tightly spread.

As illustrated graphically in Figure 3 for large errors, and Figure 4, the weighted mean of each population of spectra considered was closer to the “true” value initially constructed in the simulation than either the mean or median of the group, reflecting the underlying asymmetric shape of the ratio distribution. As can be analytically expected, this effect is much less pronounced in the case where the quantification error is smaller. For the diseased population with large errors, the mean, median, and weighted mean peak ratio values were 1.68, 1.56 and 1.60 compared to the “ground truth” value of 1.6. Under the small errors regime, these values were 1.61, 1.61 and 1.60 respectively. For the “healthy” population with large errors, these values were 2.19, 2.01 and 1.95 in comparison to the ‘ground truth’ value of 1.95; under the “small errors” regime these estimates were 1.97, 1.97 and 1.94 respectively. The sampling distribution of these quantities was also substantially less broad for the weighted mean in the population considered, when compared to the median or mean. The weighted mean, therefore, appears to recapitulate the quantity of interest in uncertain spectroscopy experiments.

**Figure 5:**
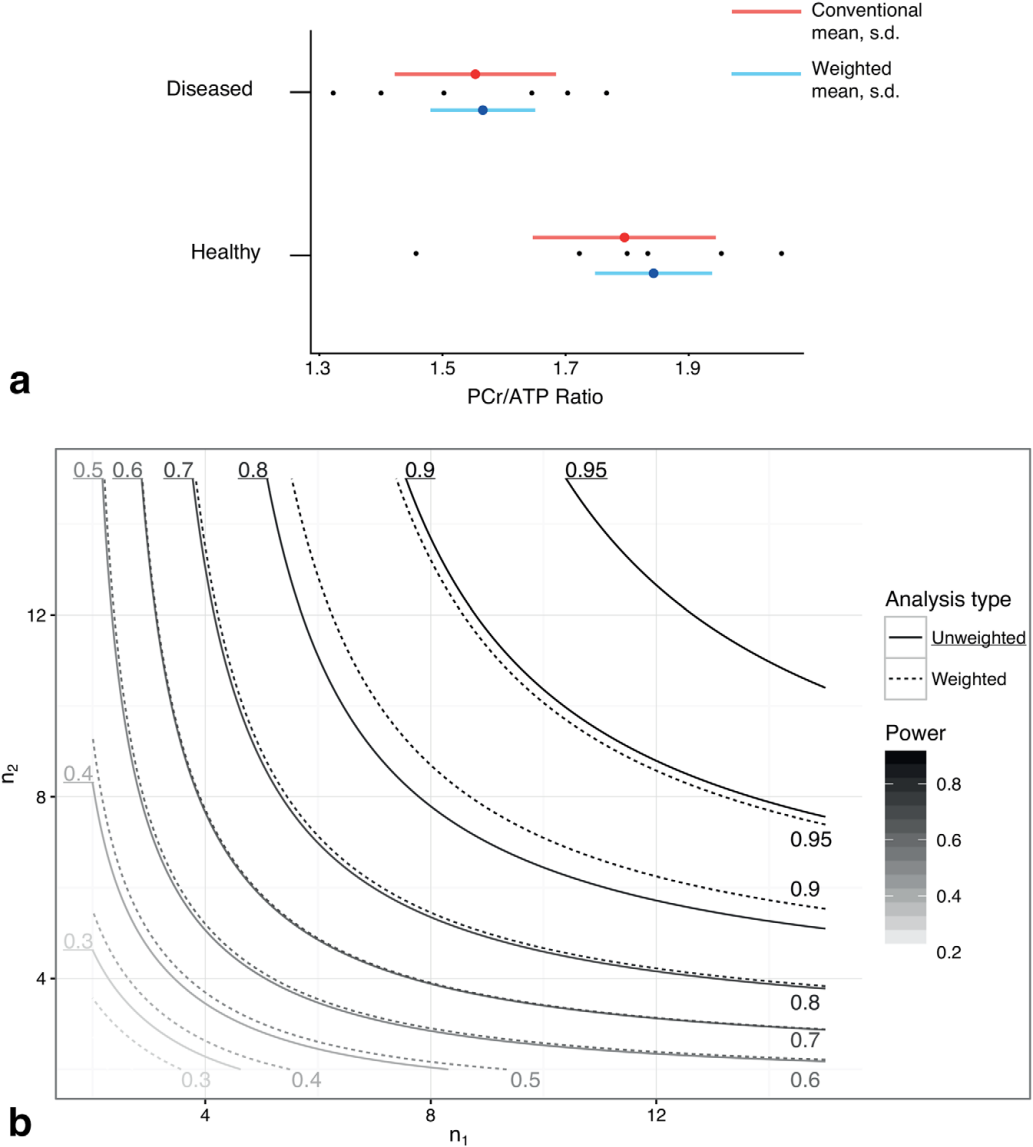
**a**: Simulated PCr/ATP ratios together with population estimates of the mean (filled circle) and standard deviation (horizontal line) for both the weighed (blue) and unweighted (red) analysis methods, together with data points (black). A reduction in the estimated population standard deviation is reflected by the use of the weighted method. **b**: Simulated statistical power for distinguishing the difference in PCr/ATP for the two populations of *n*_1_ or *n*_2_ subjects, shown as a function of *n*_1_ and *n*_2_ for both conventional (solid contours) and weighted analysis methods (broken contours). Reflecting the decrease in estimated variance, the use of a weighted analysis improves apparent statistical power.

For the representative parameter values chosen in the illustrative simulation, the difference known a priori between groups was recapitulated more effectively by correctly propagating uncertainty. The unweighted mean and standard deviation of the ‘healthy’ and ‘diseased’ groups were 1.80 ± 0.21 and 1.56 ± 0.18 respectively, compared with weighted estimates of 1.84 ± 0.04 and 1.57 ± 0.03, which is graphically illustrated in Figure 5a. Note that the value of the mean of these samples is distinct from that of the population as a whole. Accordingly, the *P* value returned from a *t*‐test between the two groups was *P* = 0.051 when data were given equal weight, or 0.024 if uncertainty was propagated as proposed.

As a direct consequence of the dramatic decrease in the estimated variance, the $g^{*}$ effect size obtained based on the data increased, from 1.519 to 1.703. Accordingly, it was possible to estimate the power of this hypothetical study as a function of the number of subjects in either group, *n*_1_ or *n*_2_, and a contour plot of statistical power as a function of *n*_1_ and *n*_2_ for both weighted and unweighted analyses methods is shown in Figure 5b. The weighted analysis method increased power for all *n*_1_ and *n*_2_, with a maximum increase of 0.098 when $n_{1}=5,\;\;n_{2}=6$ or vice versa. Therefore, neglecting variation in these observed effect sizes, a hypothetical study powered to 90% at the 0.05 significance level would require 10 individuals per group using unweighted analysis, or 8 per group with weighted averaging.

### Retrospective Analysis

#### Hyperpolarized ^13^C Spectra

**Figure 6:**
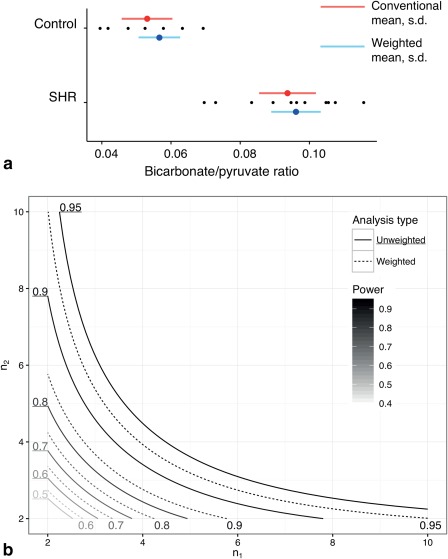
**a**: Bicarbonate‐to‐pyruvate ratios for the two populations of SHR and control rats. As in Figure 5a the weighted method reduced the apparent population variability (denoted by horizontal lines) and slightly adjusted the estimates of the mean (filled circles). **b**: A contour plot denoting the estimated power of SHR study via the weighted (broken lines) and unweighted (solid lines) analysis methods as a function of the number of animals in each group. Similarly, paralleling the simulation shown in Figure 5b, the use of weighted averages increased statistical power by a maximum of 0.05.

Accounting for the variability in the acquired hyperpolarized spectral data as proposed above improved statistical power, reduced the estimated biological variation of the population and did not appreciably change its estimated mean. As graphically illustrated in Figure 6a, the numerical effect of weighted averaging was small, largely reflecting relative homogeneity in weights. Weighted averaging reduced the population variance in SHR animals from $2.1\times 10^{-4}$ to $2.0\times 10^{-4}$, and in control animals from $1.4\times 10^{-4}$ to $1.2\times 10^{-4}$. The mean changed slightly, from 0.053 to 0.057 in control animals, and from 0.094 to 0.096 in SHR animals. Using the weighted method reduced the observed apparent variability in each population; the coefficient of variation (defined as $C_{v}={\bar{\sigma }}_{r}/\bar{r}$) was reduced from 15.36% (without incorporating uncertainty) to 14.88% (with the outlined analysis) for the SHR rat data, and from 21.24 to 20.79% for controls. As a direct consequence, the statistical power of the study would be improved had the weighted method been used throughout, with a maximal increase in power of 0.05 between the two methods occurring at $n_{1}=n_{2}=3$, as can be seen in the contour plot of statistical power (Fig. 6b). Hence, the known biochemical difference in PDH flux in the SHR rat was detected with a *P* value of $1.1\times 10^{-6}$ when error was propagated as described here, compared to $5.1\times 10^{-5}$ assuming the data had equal weight.

#### Human ^31^P Cardiac Spectra

**Figure 7:**
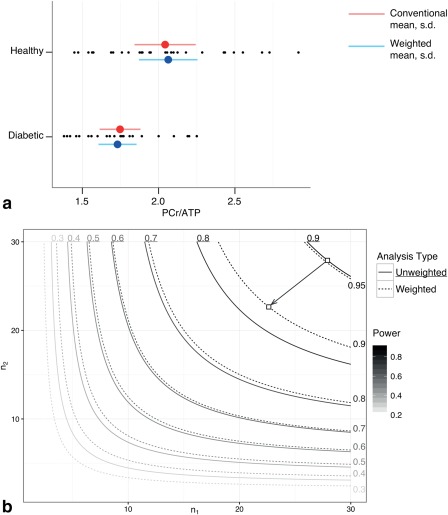
**a**: Obtained estimated cardiac PCr/ATP ratios for T2DM patients and matched controls, together with estimates of the population mean and standard deviation by both conventional and weighted approaches. The estimated biological variation in the population is reduced by the use of weighted averages; correspondingly, the estimated effect size is changed, and statistical power increases (**b**), leading to a notable reduction in the number of subjects required at the 90% power level, as illustrated.

As illustrated in Figure 7a, propagating uncertainty throughout the analysis lead to a reduction in the estimated population variation, and did not substantially change its estimated mean. The estimated mean ± standard deviation PCr/ATP ratio of T2DM patients at rest was found to be 1.747±0.268 using equal weight, or 1.730±0.249 propagating uncertainty, corresponding to a reduction in *σ* on the order of ∼7% ( $(\sigma_{\text{unweighted}}-\sigma_{\text{weighted}})/\sigma_{\text{weighted}}\approx 7\%$), and a change in the mean of less than 1%; hence *C_v_* reduced from 15.3 to 14.4%.

In contrast, the estimated mean ± standard deviation cardiac PCr/ATP of matched controls at rest was found to be 2.043±0.401 conventionally; using the weighted method produced population estimates of 2.063±0.384, again resulting in a small ( <0.25%) alteration to the position of the mean but a 4% reduction in *σ*, and a corresponding change in the *C_v_* from 19.6 to 18.6%.

Consequently, the estimated effect size and hence statistical power of the study improved, with a change in $g^{*}$ from 0.884 to 0.985 between the two methods, as is illustrated in Figure 7b. As a corollary, the *P* value obtained from an (unpaired, Welch's) *t*‐test between the two groups reduced from 0.0031 to 0.00058 by the use of weighted averaging. Additionally, a proposed study investigating this difference with power 0.9 at a significance level of 0.05 would require 28 individuals in each group with the unweighted analysis method, or 23 using weighted averaging.

## DISCUSSION

These short illustrative examples have demonstrated the practical need to propagate uncertainty in in vivo spectroscopic studies in which the quantification error on peak amplitudes should be used. The analysis approach proposed here effectively allows for the reduction of measurement uncertainty in the estimation of variation of the population being studied, leading to a reduction in the estimated variance of simulated PCr/ATP data, and yielded gains in two representative, and very distinct, biological examples. Accounting for variation in spectral data with weights inferred from quantification uncertainty, therefore, represents a conceptually straightforward way to improve the estimation of the value of a parameter in the population from which those measurements were obtained.

In vivo MRS suffers from inherent SNR limitations, and the analysis of spectra possessing low or variable SNR remains an important issue. The competing requirements of scan‐times short enough to be tolerable for subjects and yet providing sufficient SNR to quantify small metabolite peaks will fundamentally lead to spectral data that has varying, comparatively low, SNR. Low SNR corresponds to greater quantification errors, and directly affects the shape of the resulting distribution of values obtained. In the case of ^31^P, low SNR has resulted in a broad range of published values for many quantities, such as the healthy cardiac PCr/ATP ratio as determined by ^31^P MRS, which is widely regarded to be “around two” 29. As originally noted by Paul Bottomley in 1992 30, this variability has led historically to reproducibility problems whereby systematic biases and poor spectral quantification pipelines led independent groups to experimentally support diametrically opposed conclusions in the early 1990s. It is for this reason that maximum acceptable linewidth criteria and SNR thresholds (typically of 10) are commonly proposed 31.

The importance of rigorous statistical analysis of metabolite ratios is far from unrecognized in MRS; Bottomley himself notes that “[…] one expects to see random scatter in measurements of metabolite concentrations or ratios that is commensurate with the particular [SNR] of the moieties in question”, and that “If the error is truly random, a careful statistical analysis of many such measurements from different study groups (preferably blinded) can reveal a significant scientific or even clinically useful finding” 30. Despite this early message, quantification uncertainty is not usually propagated accurately when metabolite ratios are considered, although weighted averaging is routinely used in other physical sciences. Additionally, note that the problem of combining uncertain data from different subjects is directly analogous to that of recombining multi‐coil spectroscopy data, for which it is well known that the use of weights that are the square of the estimated SNR in each spectrum are optimal 32. The method proposed here is directly analogous to using such techniques at the population level.

A valid criticism of using weighted averages in in vivo spectroscopic studies is that it is not necessarily the case that the least certain data are the least accurate: a noiseless measurement of a systemically flawed experiment is given greater weight in the above analysis than that of a noisy, but unbiased, one. There are many factors affecting the scan‐to‐scan SNR in MR spectroscopy, such as coil placement and loading differences between subjects. A priori, it therefore seems plausible that a reduction in the acquisition SNR occurs on physical grounds, and does indeed represent an experimental deviation of that particular measurement from the mean. It is worthy of note, however, that the ratio distribution is a five‐parameter distribution and changes in peak ratios can occur due to changes in either the numerator or denominator: owing to the difference in quantification uncertainty, these two scenarios are distinct, but could be estimated analytically given parameter values. Likewise, the distinctly non‐normal shape of the ratio distribution with highly uncertain peaks would indicate that caution should be used when performing parametric statistical tests between populations.

The difference in behavior of sets of weighted averages from analysis of the same data using the unweighted mean are well known, and are often encapsulated in scenarios such as Simpson's paradox, originally framed as a problem in which the gender bias in a university's admission process could be misrepresented in either direction by the inappropriate use of unweighted averages 33. In MR, it is proposed that the appropriate ‘data driven’ approach is to trust the information contained in the variability of data, and consider weighted analysis, potentially in addition to the conventional, unweighted approach. For the biological examples considered here, the reduction in estimated variance by the use of weighted averages is at the very least consistent with this assumption. Note that the selective elimination of spectra with low SNR has recently been shown to lead to bias in the context of population NMR studies, via the distortion of the sampling distribution 34 which this technique may potentially ameliorate, particularly when the distribution of quantities considered is approximately normal (i.e. peak sums, or ratios where the distribution is approximately normal).

Whilst emerging techniques, such as hyperpolarization, offer a way to improve the inherent SNR limitations of magnetic resonance, small variations in the timing of experiments can have a large impact in the resulting quality of the data acquired, leading again to the consideration of this problem in a different context. As information regarding reproducibility is of great importance to the design and analysis of clinical studies, ensuring that experiments are statistically maximally empowered is of great value. In the above example analyses, under ideal circumstances the use of weighted averages would allow for an increase of ∼5 to 10% in statistical power. In a preclinical setting, this increase could be directly traded for a reduction in the number of experimental animals required to reach the same conclusion. Properly propagating uncertainty is therefore ethically favored. In clinical settings, an increase in power and the ability to appropriately include spectra that do not meet an arbitrary SNR threshold would both likely decrease the amount of scan time required by any individual study, potentially improve patient compliance, and therefore present the potential for a significant economic saving.

## CONCLUSIONS

It is proposed that the study‐level adoption of methods that incorporate estimates of uncertainty, such as described here, will allow the maximal use of data that has been acquired with varying precision. The proposed analysis is conceptually and computationally simple and offers immediate value to existing data, as illustrated by a retrospective analysis undertaken on previously published results. Weighted versions of several (but by no means all) common statistical techniques are readily available in numerous popular programming languages, and most spectral quantification algorithms return estimates of uncertainty on their results. The improved statistical power provided by the correct use of weighted averaging may help reveal small biological differences between populations of measurements, or avoid false conclusions.

## Supporting information

Supporting Information

## Appendix

The analytic form of the ratio distribution (i.e. the distribution of the ratio of two normally distributed random variables) is not straightforward, and is analytically provided as a function of the ratio, w=x/y where *x* and *y* have a correlation coefficient *ρ*, by the expression:

$$f(w)=\frac{b(w)d(w)}{\sqrt{2\pi }\sigma_{x}\sigma_{y}a^{3}(w)}\left(\Phi \left(\frac{b(w)}{\sqrt{1-\rho^{2}}a(w)}\right)-\Phi \left(-\frac{b(w)}{\sqrt{1-\rho^{2}}a(w)}\right)\right)$$

(A1) $$+\frac{\sqrt{1-\rho^{2}}}{\pi \sigma_{x}\sigma_{y}a^{2}(w)}\exp\left(-\frac{c}{2(1-\rho^{2})}\right),$$

where

(A2) $$a(w)={\left(\frac{w^{2}}{\sigma_{x}^{2}}-\frac{2\rho w}{\sigma_{x}\sigma_{y}}+\frac{1}{\sigma_{y}^{2}}\right)}^{\frac{1}{2}}$$

(A3) $$b(w)=\frac{\mu_{x}w}{\sigma_{x}^{2}}-\frac{\rho (\mu_{x}+\mu_{y}w)}{\sigma_{x}\sigma_{y}}+\frac{\mu_{y}}{\sigma_{y}^{2}}$$

(A4) $$c=\frac{\mu_{x}^{2}}{\sigma_{x}^{2}}+\frac{2\rho \mu_{x}\mu_{y}}{\sigma_{x}\sigma_{y}}+\frac{\mu_{y}^{2}}{\sigma_{y}^{2}}$$

(A5) $$d(w)=\exp\left(\frac{b^{2}(w)-c(w)a^{2}(w)}{2(1-\rho^{2})a^{2}(w)}\right);$$

and

$$\Phi (z)=\frac{1}{\sqrt{2\pi }}\int_{-\infty }^{z}e^{-\frac{1}{2}u^{2}}\;du$$

(A6) $$=\frac{1}{2}\left(1+\text{Erf}\left(\frac{z}{\sqrt{2}}\right)\right),$$

where Erf(z) denotes the Gaussian error function, $\text{Erf}(z)=\frac{2}{\sqrt{\pi }}\int_{0}^{z}e^{-t^{2}}\;dt$, and $w=\frac{x∼N(\mu_{x},\;\sigma_{x})}{y∼N(\mu_{y},\;\sigma_{y})}$.
