# Supplementary material for: Weighted averaging in spectroscopic studies improves statistical power
Source: Magn Reson Med. 2017 Jan 26;78(6):2082–94. doi: 10.1002/mrm.26615 (PMC5697704; doi:10.1002/mrm.26615)
Supplement: Supplementary file 1 — Supporting Information [file MRM-78-2082-s001.pdf]

Online Supporting Material: Derivation of the Weighted Mean and Variance

For the sake of completeness, a brief derivation of weighted expressions for mean and variance are presented, as it is difficult to find satisfactory equivalents in the literature. As discussed in the Theory section of the manuscript, spectral fitting algorithms frequently return a quantity of interest from a spectral peak μ_i, (e.g. its amplitude) additionally with an estimate of uncertainty of that quantity (e.g. the CRLB), σ_i. Each measurement of μ_i may be considered to be independent and identically distributed. As noise in MR is white, it is justified to treat each of these results as being normally randomly distributed variables x_i, with $P(x_i) = \mathcal{N}(\mu_i,\, \sigma_i)$. If we have n such measurements, then the joint probability distribution function (PDF) defined is simply the product of each PDF, i.e.:

$$P(x_1, x_2, \cdots, x_n) = \prod_{i=1}^{n} \mathcal{N}(\mu_i, \sigma_i) = \prod_{i=1}^{n} \frac{1}{\sqrt{2\pi\sigma_i}} \exp\left(-\frac{1}{2}\frac{(\mu_i - \mu)^2}{\sigma_i^2}\right). \tag{1}$$

where μ is the overall mean of the population we wish to identify. It is therefore the case that we wish to maximise the likelihood of obtaining μ, i.e. differentiate P with respect to μ, and solve that equal to zero:

$$\frac{\partial}{\partial\mu} \sum_i \left(-\frac{1}{2}\frac{(\mu_i - \mu)^2}{\sigma_i^2}\right) = \sum_i \left(\frac{\mu_i - \mu}{\sigma_i^2}\right) = 0 \tag{2}$$

$$\Rightarrow \mu = \frac{\sum_i^n \frac{\mu_i}{\sigma_i^2}}{\sum_i^n \frac{1}{\sigma_i^2}} \tag{3}$$

$$\tag{4}$$

i.e. we take a weighted mean, where each weight w_i is the reciprocal variance of each PDF, $w_i = 1/\sigma_i^2$, normalised by the sum of the weights $W = \sum_i w_i$:

$$\bar{x} = \frac{1}{W} \sum_i w_i x_i.$$

Additionally, we desire an unbiased estimator of the variance of the underlying distribution, σ^2. That is, we wish to identify a formula for the weighted variance (or standard deviation) as well as the mean.

In analogy with the method for computing Bessel's correction for the sample variance, (that is, the corrective factor of $N - 1$ in the denominator) we recall the standard definition of the sample variance V and compute its expectation. We then infer a bias correction factor, and, hence, an unbiased weighted variance estimator.

$$
\begin{aligned}
V &\stackrel{\text{def}}{=} \frac{1}{W}\sum_i w_i(x_i-\bar{x})^2 \\
&= \frac{1}{W}\sum_i w_i\left(x_i - \frac{1}{W}\sum_j w_j x_j\right)^2 \\
&= \frac{1}{W}\sum_i w_i\left(x_i^2 - \frac{2}{W}x_i\sum_j w_j x_j + \frac{1}{W^2}\left(\sum_j w_j x_j\right)^2\right) \\
&= \frac{1}{W}\left(\sum_i w_i x_i^2 - \frac{1}{W}\sum_i w_i x_i \sum_j w_j x_j\right) \\
&= \frac{1}{W}\left(\sum_i w_i x_i^2 - \frac{1}{W}\sum_{ij} w_i w_j x_i x_j\right) \qquad (5)
\end{aligned}
$$

We now compute the expectation value for this estimator, $\langle V\rangle$. Recall that, for normally distributed random variables, $\langle x_i\rangle = \mu$, and $\langle x_i x_j\rangle = \mu^2 + \delta_{ij}\sigma^2$. We additionally assume that any random contribution dependent on the weights themselves is zero or can be neglected in comparison to σ, such that $\langle w_i x_i\rangle = w_i\langle x_i\rangle$ and $\langle w_i w_j x_i x_j\rangle = w_i w_j\langle x_i x_j\rangle$.

$$
\begin{aligned}
\langle V\rangle &= \frac{1}{W}\left(\sum_i w_i\langle x_i^2\rangle - \frac{1}{W}\sum_{ij} w_i w_j\langle x_i x_j\rangle\right) \\
&= \frac{1}{W}\left(\sum_i w_i(\mu^2+\sigma^2) - \frac{1}{W}\sum_{ij} w_i w_j\left(\mu^2+\delta_{ij}\sigma^2\right)\right) \\
&= \frac{1}{W}\left(W(\mu^2+\sigma^2) - \frac{1}{W}(W^2\mu^2 + (\sum_i w_i^2)\sigma^2)\right) \\
&= \frac{1}{W}\left(W\sigma^2 - \frac{1}{W}(\sum_i w_i^2)\sigma^2\right) \\
&= \left(\frac{W^2 - \sum_i w_i^2}{W^2}\right)\sigma^2 \qquad (6)
\end{aligned}
$$

Therefore an unbiased estimator U of σ^2 is

$$
\begin{aligned}
U &= \frac{W^2}{(W^2 - \sum_i w_i^2)}\langle V\rangle \\
&= \frac{W^2}{(W^2 - \sum_i w_i^2)}\frac{1}{W}\sum_i w_i(x_i-\bar{x})^2 \\
&= \frac{W}{(W^2 - \sum_i w_i^2)}\sum_i w_i(x_i-\bar{x})^2 \qquad (7)
\end{aligned}
$$

which is identical to Eq. (6) in the main manuscript, where the weights have been stated explicitly, i.e. where $w_i = 1/\sigma_i^2$.

This expression has several reassuring properties: it is invariant under multiplicative weight transformation; and in the case that all weights are equal, $w_i = w$, say, the pre-multiplying factor reduces to $N/(N^2-N) = 1/(N-1)$, which is the same pre-multiplying factor for the familiar expression of an unbiased estimator of variance from data with equal uncertainty.
